# Supplementary material for: Evaluation of Polyphenols Synthesized in Mature Seeds of Common Bean (Phaseolus vulgaris L.) Advanced Mutant Lines
Source: Int J Mol Sci. 2024 May 22;25(11):5638. doi: 10.3390/ijms25115638 (PMC11172207; doi:10.3390/ijms25115638)
Supplement: Supplementary file 1 [file ijms-25-05638-s001.zip › ijms-2959230-supplementary.pdf]

Supplementary material

Evaluation of anthocyanins synthesized in mature seeds of common bean (*Phaseolus Vulgaris* L.) advanced mutant lines

Teodora G. Yaneva[1]*, Wieslaw Wiczkowski[2], Andrey S. Marchev[3], Dida Zh. Iserliyska[1], Milen I. Georgiev[3], Nasya B. Tomlekova[4]*

[1]Department of Food Technology, Institute of Food Preservation and Quality, Agricultural Academy, 154 Vasil Aprilov Blvd., 4027 Plovdiv, Bulgaria

[2]Department of Chemistry and Biodynamics of Food, Institute of Animal Reproduction and Food Research, Polish Academy of Sciences, 10 Tuwima Str., 10-748 Olsztyn, Poland

[3]Laboratory of Metabolomics, Department of Biotechnology, Stephan Angeloff Institute of Microbiology, Bulgarian Academy of Sciences, 139 Ruski Blvd., 4000 Plovdiv, Bulgaria

[4]Laboratory of Molecular Biology, Department of Breeding, Maritsa Vegetable Crops Research Institute, Agricultural Academy, 32 Brezovsko shosse Str., 4003 Plovdiv, Bulgaria

***Correspondence:**

Teodora G. Yaneva; t.yaneva@canri.org
Nasya B. Tomlekova; nasia.tomlekova@gmail.com

Keywords: Common bean (*Phaseolus vulgaris* L.); anthocyanins; seed color; HPLC-MS/MS; EMS-induced mutagenesis

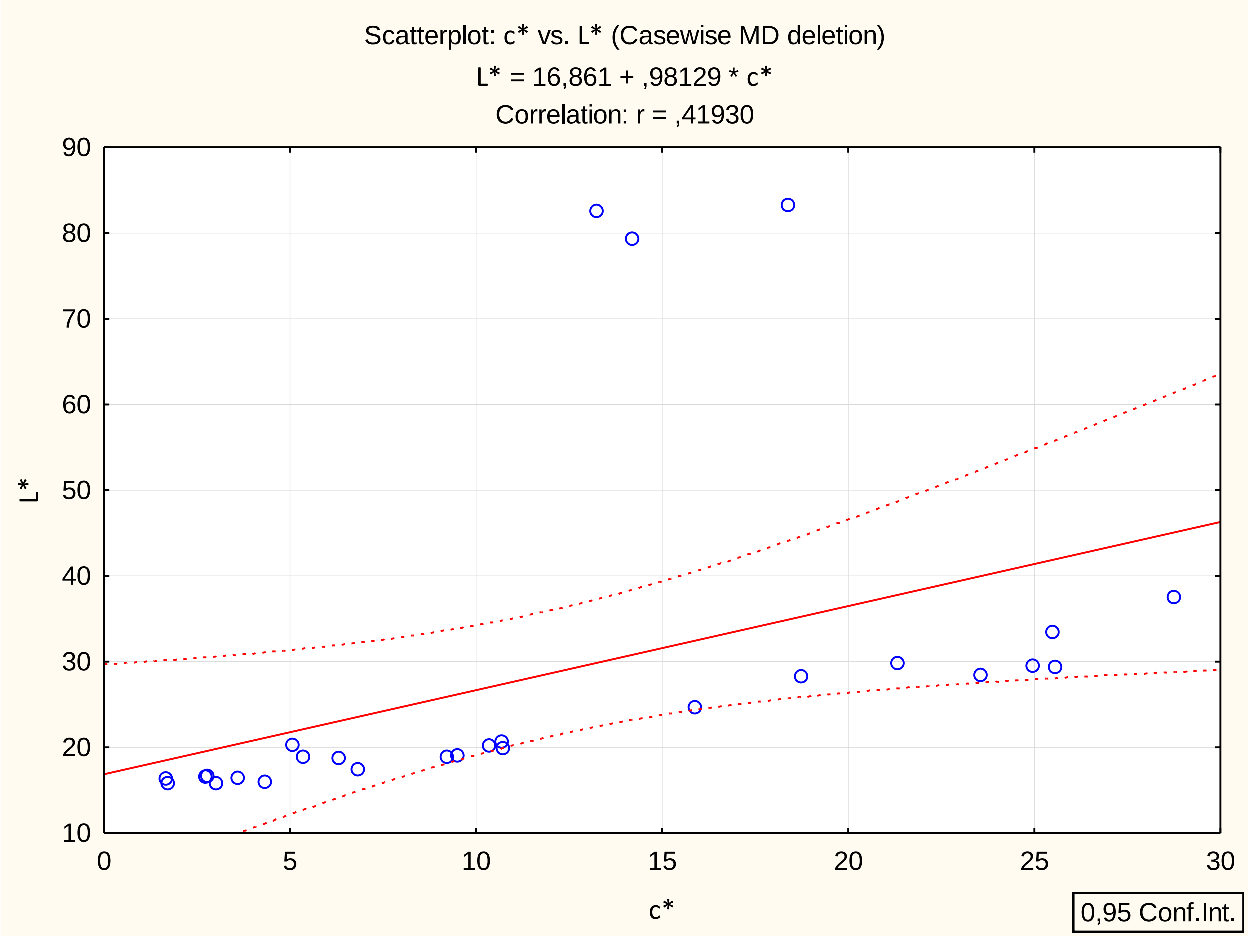

Figure S1. Correlation of L and C (color parameters) of 27 common bean genotypes. L – illuminance (L=0 – black, L=100 – white), +a – red color, -a – green color, +b – yellow color, -b – blue color.

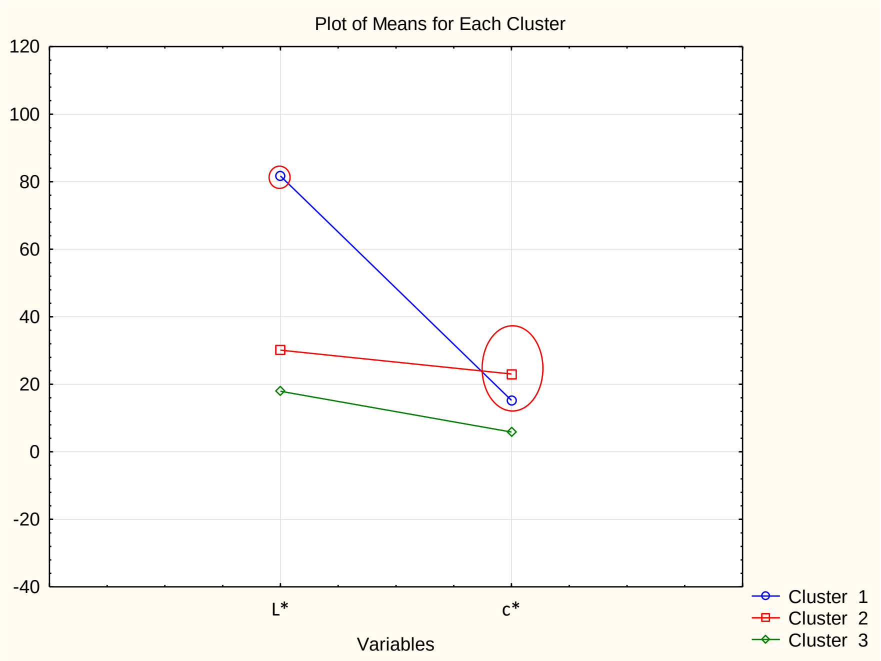

Figure S2. Clusters mean for luminosity (L) and chroma (C) in 27 common bean genotypes

Table S1. Colorimetric parameters of 25 dry bean mutant genotypes, the initial and a local cultivar.

CIE lab		L*	a*	b*	c*	h*	WI	dE*
mutant line	Genotype	Brightness	Red/Green	Blue/Yellow	Chroma	Hue	Whiteness/CIELAB	
control		96.94	0.32	0.49	0.58	56.85	92.16	
M 2.1	M 564-56-4-3-1	79.34	2.33	13.99	14.19	80.52	46.67	22.31
M 2.2	M 564-56-4-3-2	29.82	12.13	17.52	21.33	55.21	4.09	70.33
M 2.3	M 564-56-4-3-3	16.39	2.15	2.82	3.61	56.11	3.02	80.64
M 3	M 564-110-1	82.51	1.97	13.09	13.24	81.52	49.07	19.33
M 6.1	M 564-172-2-1-1	24.61	10.07	12.19	15.88	49.87	3.60	74.06
M 6.2	M 564-172-2-1-2	18.72	4.15	4.74	6.32	48.64	3.26	78.48
M 7.1	M 564-175-3-1-1	28.43	14.90	18.10	23.56	49.58	3.71	72.69
M 7.2	M 564-175-3-1-2	19.85	7.58	7.49	10.73	43.21	3.18	77.89
M 7.3	M 564-175-3-1-3	17.41	4.91	4.67	6.83	45.84	3.03	82.03
M 8.1	M 564-175-3-3-1	29.51	16.79	18.39	24.96	47.49	3.91	71.75
M 8.2	M 564-175-3-3-2	19.05	7.53	5.74	9.50	36.31	3.22	78.46
M 8.3	M 564-175-3-3-3	15.77	1.33	1.06	1.72	42.09	3.10	81.20
M 9.1	M 564-175-3-4-1	18.83	6.56	6.40	9.23	44.64	3.09	78.67
M 9.2	M 564-175-3-4-2	16.54	2.01	1.82	2.74	48.70	3.16	80.45
M 10	M 564-190-1-1	33.43	15.05	20.52	25.49	53.53	4.61	68.35
M 11.1	M 564-190-3-1-1	37.47	15.84	23.97	28.75	56.44	5.15	65.90
M 11.2	M 564-190-3-1-2	20.66	6.70	8.28	10.70	50.37	3.25	77.04
M 11.3	M 564-190-3-1-3	15.97	2.93	3.17	4.33	46.27	2.91	81.08

M 12	M 564-190-3-2	16.64	2.24	1.53	2.78	45.54	3.22	80.36
M 14	M 564-190-3-4	20.29	3.73	3.21	5.08	52.61	3.87	76.87
M 15	M 564-190-3-5	16.32	1.56	0.30	1.66	105.72	3.28	78.62
M 16.1	M 564-190-3-7-1	n/a	n/a	n/a	n/a	n/a	n/a	n/a
M 16.2	M 564-190-3-7-2	18.89	4.09	3.38	5.36	37.71	3.45	78.24
M 17	M 564-191-1-1	15.76	2.46	1.54	3.01	55.11	3.05	81.27
M 19.1	M 564-193-9-1-1	29.32	16.67	17.95	25.58	48.48	3.76	72.41
M 19.2	M 564-193-9-1-2	20.20	7.19	7.21	10.35	49.22	3.29	78.27
Local cv.	“Tangra”	28.30	12.00	14.33	18.74	49.38	4.19	71.13
Initialcv.	“Evros”	83.24	3.63	18.02	18.38	78.68	45.95	22.69

The indicators were determined according to the CIE Lab system, where: L - luminosity (L = 0 - black, L = 100 - white), +a - red color, -a - green color, +b - yellow color, -b - blue color

Table S2. V-fold cross tabulation table of the variable bean genotype

	Summary Frequency Table. Marked cells have counts > 10 (Marginal summaries are not marked)									
	Beans	Final classification 1	Final classification 2	Final classification 3	Final classification 4	Final classification 5	Final classification 6	Final classification 7	Final classification 8	Final classification 9	Row Totals
Count	M 2.1	0	14	0	0	0	0	0	0	0	14
Row Percent		0.00%	100.00%	0.00%	0.00%	0.00%	0.00%	0.00%	0.00%	0.00%	
Count	M 2.2	0	0	0	0	3	0	0	18	4	25
Row Percent		0.00%	0.00%	0.00%	0.00%	12.00%	0.00%	0.00%	72.00%	16.00%	
Count	M 2.3	20	0	2	29	0	0	0	0	0	51
Row Percent		39.22%	0.00%	3.92%	56.86%	0.00%	0.00%	0.00%	0.00%	0.00%	
Count	M 3	0	42	0	0	0	4	0	0	0	46
Row Percent		0.00%	91.30%	0.00%	0.00%	0.00%	8.70%	0.00%	0.00%	0.00%	
Count	M 6.1	0	0	19	0	1	0	0	9	29	58
Row Percent		0.00%	0.00%	32.76%	0.00%	1.72%	0.00%	0.00%	15.52%	50.00%	
Count	M 6.2	37	0	14	11	0	0	0	0	1	63
Row Percent		58.73%	0.00%	22.22%	17.46%	0.00%	0.00%	0.00%	0.00%	1.59%	
Count	M 7.1	5	0	1	0	17	0	8	23	0	54
Row Percent		9.26%	0.00%	1.85%	0.00%	31.48%	0.00%	14.81%	42.59%	0.00%	

Count	M 7.2	12	0	22	0	0	0	0	2	7	43
Row Percent		27.91%	0.00%	51.16%	0.00%	0.00%	0.00%	0.00%	4.65%	16.28%	
Count	M 7.3	12	0	1	31	0	0	0	0	0	44
Row Percent		27.27%	0.00%	2.27%	70.45%	0.00%	0.00%	0.00%	0.00%	0.00%	
Count	M 8.1	0	0	0	0	29	0	0	26	0	55
Row Percent		0.00%	0.00%	0.00%	0.00%	52.73%	0.00%	0.00%	47.27%	0.00%	
Count	M 8.2	5	0	21	1	0	0	0	0	2	29
Row Percent		17.24%	0.00%	72.41%	3.45%	0.00%	0.00%	0.00%	0.00%	6.90%	
Count	M 8.3	6	0	0	61	0	0	0	0	0	67
Row Percent		8.96%	0.00%	0.00%	91.04%	0.00%	0.00%	0.00%	0.00%	0.00%	
Count	M 9.1	9	0	14	2	0	0	0	0	3	28
Row Percent		32.14%	0.00%	50.00%	7.14%	0.00%	0.00%	0.00%	0.00%	10.71%	
Count	M 9.2	12	0	1	40	0	0	0	0	0	53
Row Percent		22.64%	0.00%	1.89%	75.47%	0.00%	0.00%	0.00%	0.00%	0.00%	
Count	M 10	0	0	0	0	43	0	13	28	4	88
Row Percent		0.00%	0.00%	0.00%	0.00%	48.86%	0.00%	14.77%	31.82%	4.55%	
Count	M 11.1	0	0	0	0	9	0	34	3	1	47
Row Percent		0.00%	0.00%	0.00%	0.00%	19.15%	0.00%	72.34%	6.38%	2.13%	
Count	M 11.2	10	0	27	0	0	0	0	0	6	43
Row Percent		23.26%	0.00%	62.79%	0.00%	0.00%	0.00%	0.00%	0.00%	13.95%	
Count	M 11.3	28	0	2	20	0	0	0	0	0	50
Row Percent		56.00%	0.00%	4.00%	40.00%	0.00%	0.00%	0.00%	0.00%	0.00%	
Count	M 12	19	0	1	46	0	0	0	0	0	66
Row Percent		28.79%	0.00%	1.52%	69.70%	0.00%	0.00%	0.00%	0.00%	0.00%	
Count	M 14	33	0	14	32	0	0	0	0	2	81
Row Percent		40.74%	0.00%	17.28%	39.51%	0.00%	0.00%	0.00%	0.00%	2.47%	
Count	M 15	3	0	0	68	0	0	0	0	0	71
Row Percent		4.23%	0.00%	0.00%	95.77%	0.00%	0.00%	0.00%	0.00%	0.00%	
Count	M 16.2	47	0	11	12	0	0	0	0	0	70
Row Percent		67.14%	0.00%	15.71%	17.14%	0.00%	0.00%	0.00%	0.00%	0.00%	

Count	M 17	12	0	6	42	0	0	0	0	0	60
Row Percent		20.00%	0.00%	10.00%	70.00%	0.00%	0.00%	0.00%	0.00%	0.00%	
Count	M 19.1	0	0	0	0	12	0	11	12	7	42
Row Percent		0.00%	0.00%	0.00%	0.00%	28.57%	0.00%	26.19%	28.57%	16.67%	
Count	M 19.2	17	0	6	16	0	0	0	0	2	41
Row Percent		41.46%	0.00%	14.63%	39.02%	0.00%	0.00%	0.00%	0.00%	4.88%	
Count	Local cultivar	0	0	2	0	3	0	0	12	22	39
Row Percent		0.00%	0.00%	5.13%	0.00%	7.69%	0.00%	0.00%	30.77%	56.41%	
Count	Initial cultivar	0	6	0	0	0	39	0	0	0	45
Row Percent		0.00%	13.33%	0.00%	0.00%	0.00%	86.67%	0.00%	0.00%	0.00%	
Count	All Groups	287	62	164	411	117	43	66	133	90	1373

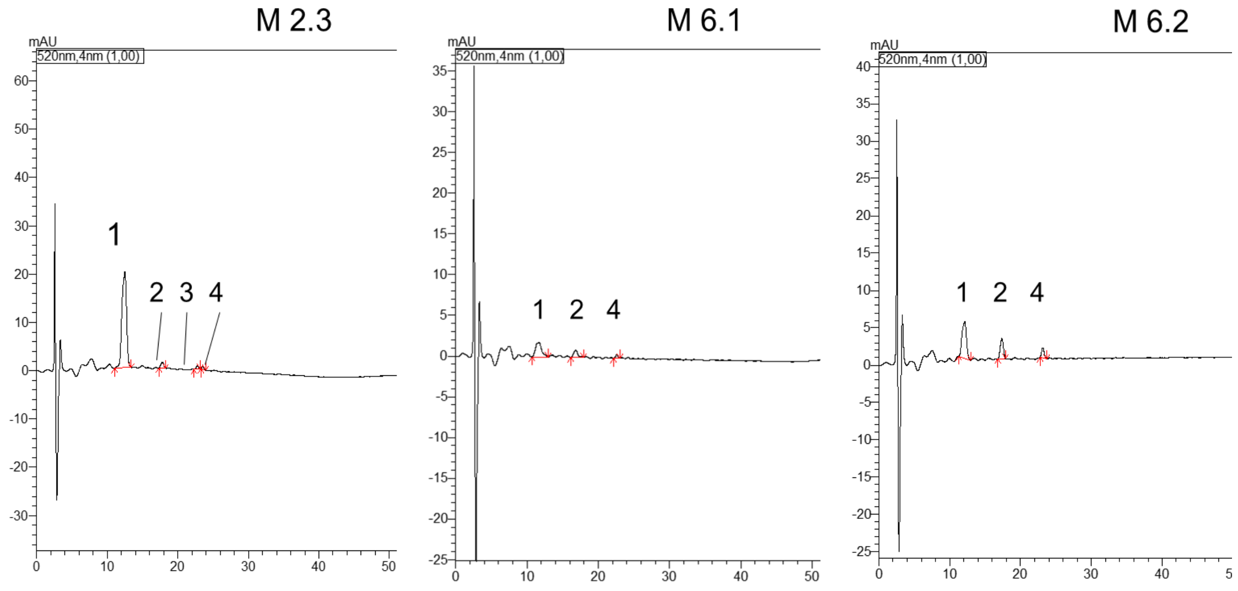

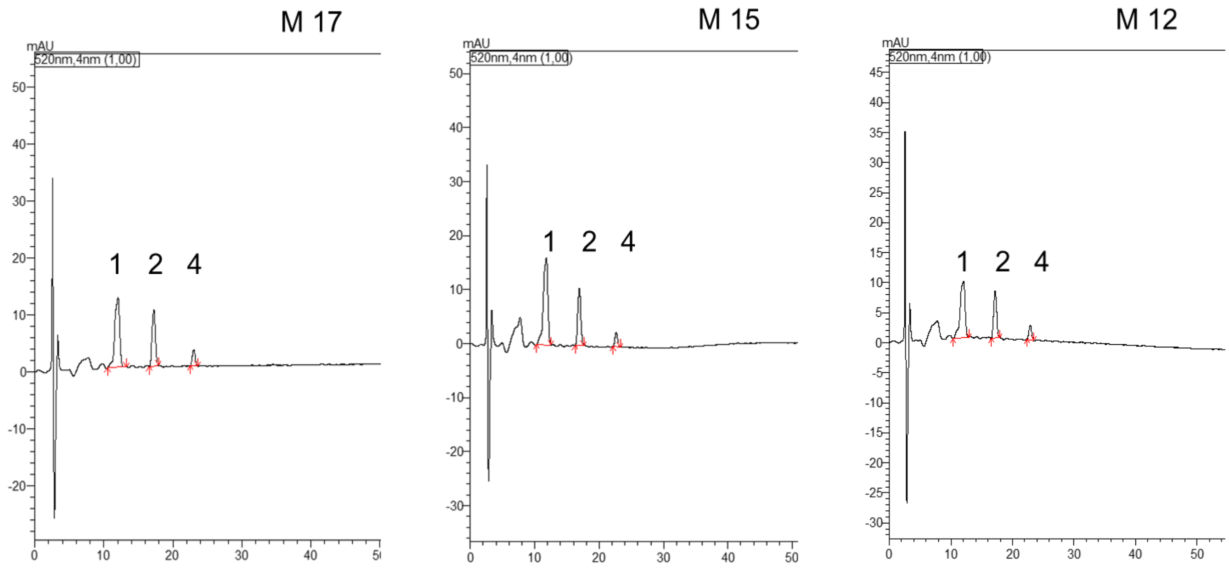

Figure S3. Retention time of the anthocyanins in common bean extract Chromatograms of extracts of mutant bean lines of M 2.3; M 6.1; M 6.2, M 17; M 15; M 12 at 520 nm.

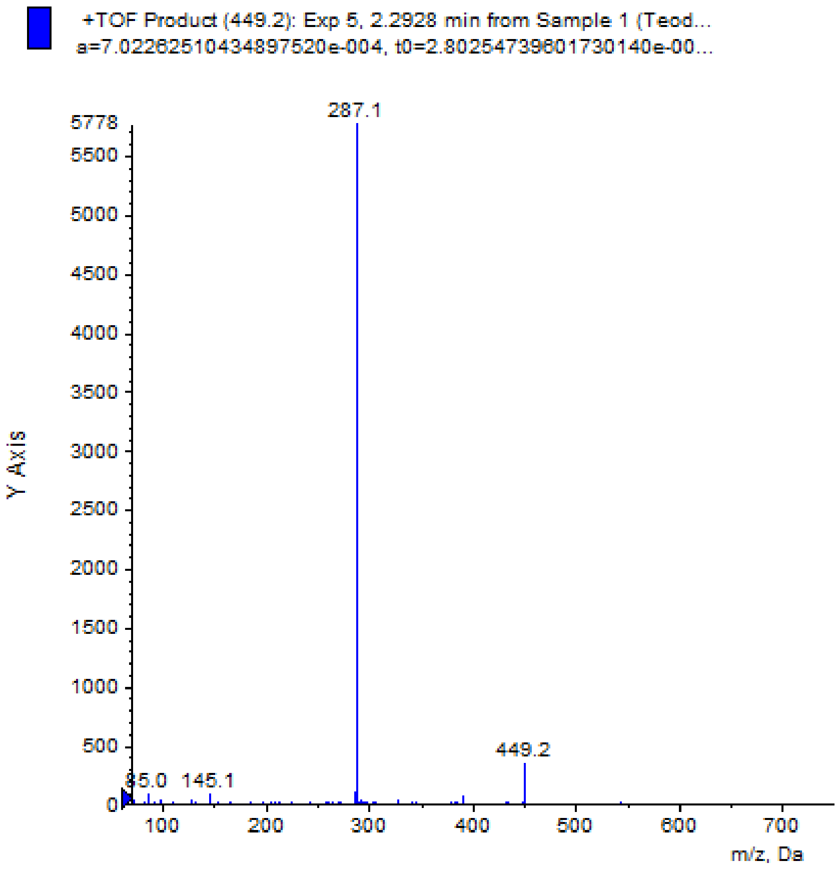

Cyanidin 3-glucoside in M 2.3

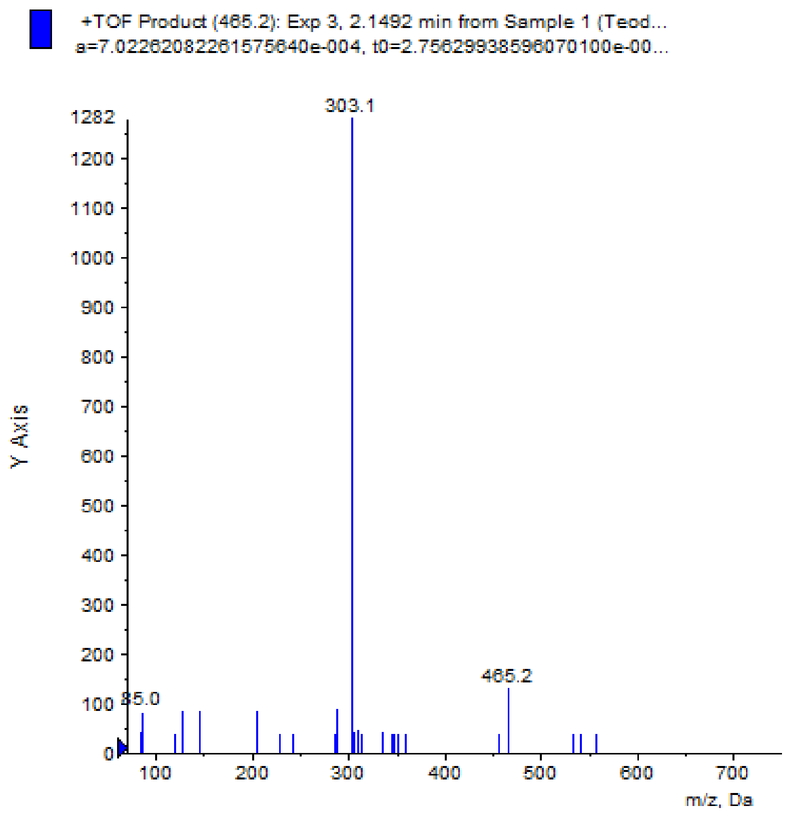

Delphinidin 3-glucoside in M 2.3

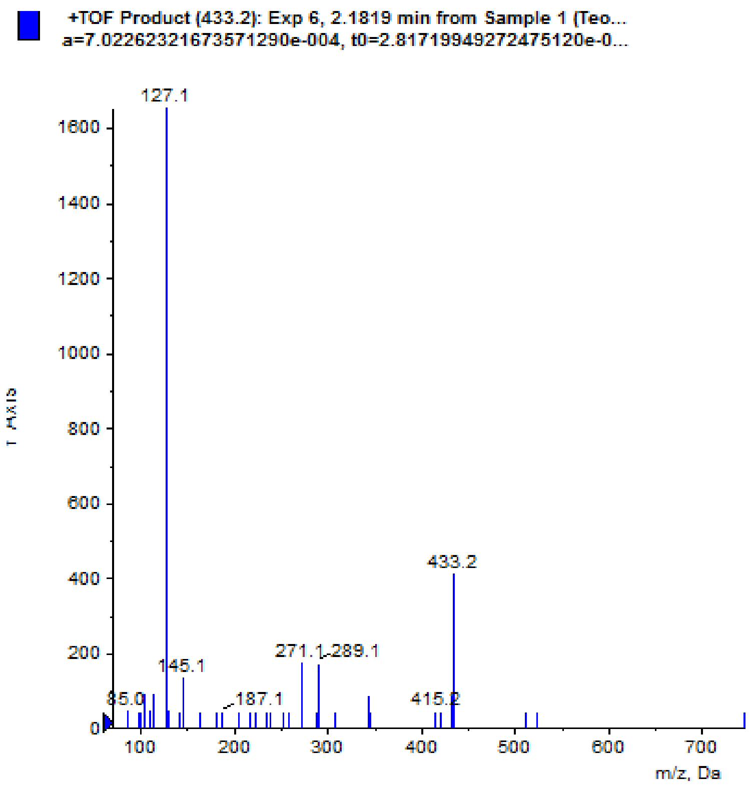

Pelargonidin 3-glucoside in M.2.3

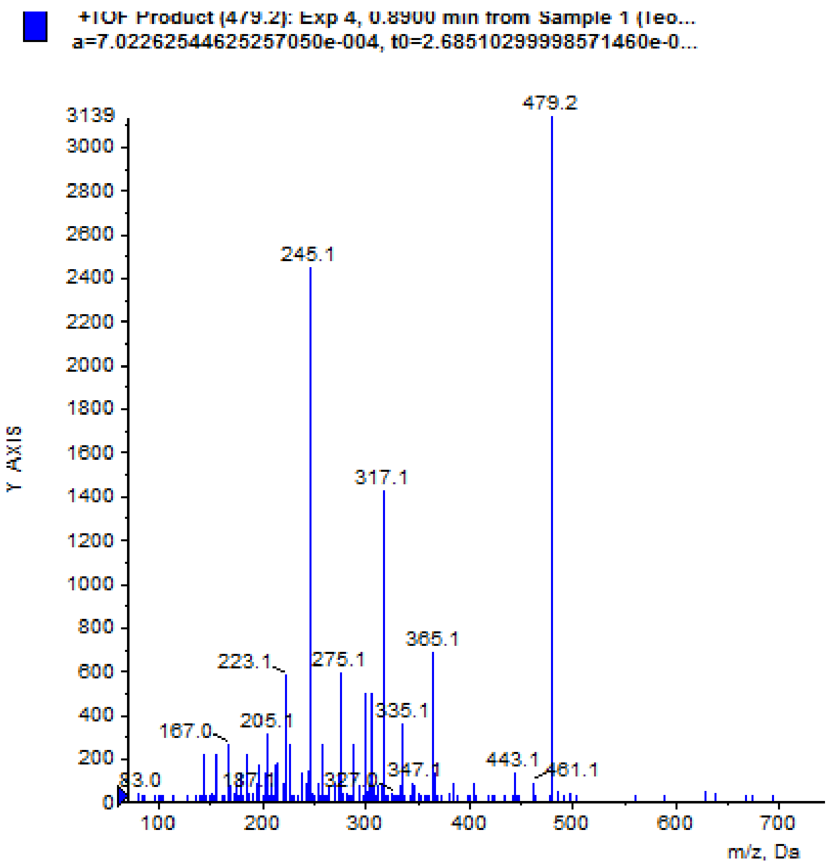

Petinidin 3-glucoside in M 2.3

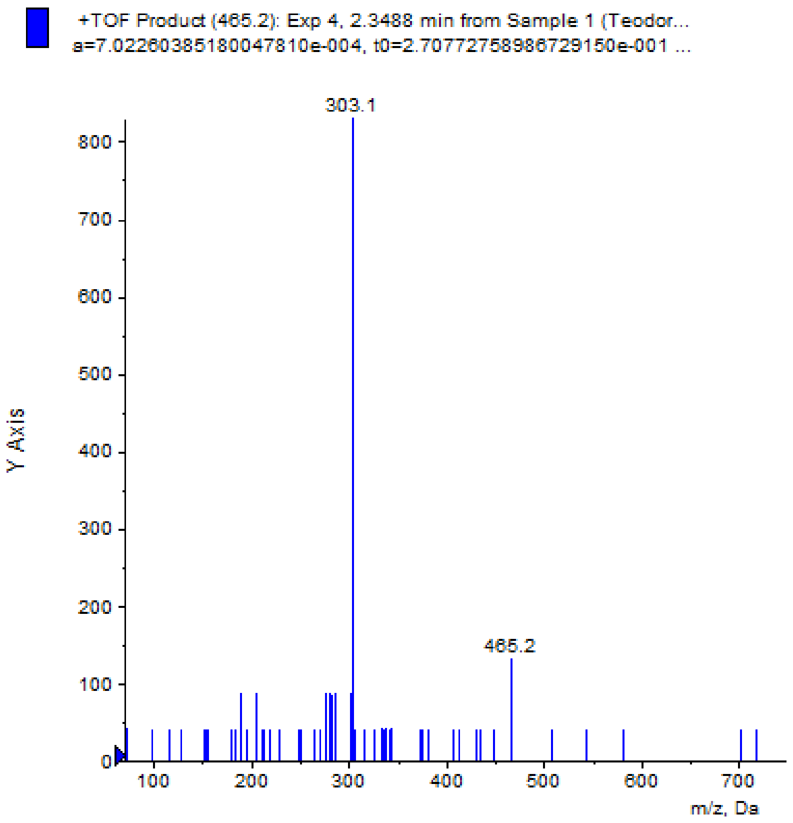

Cyanidin 3-glucoside in M 15

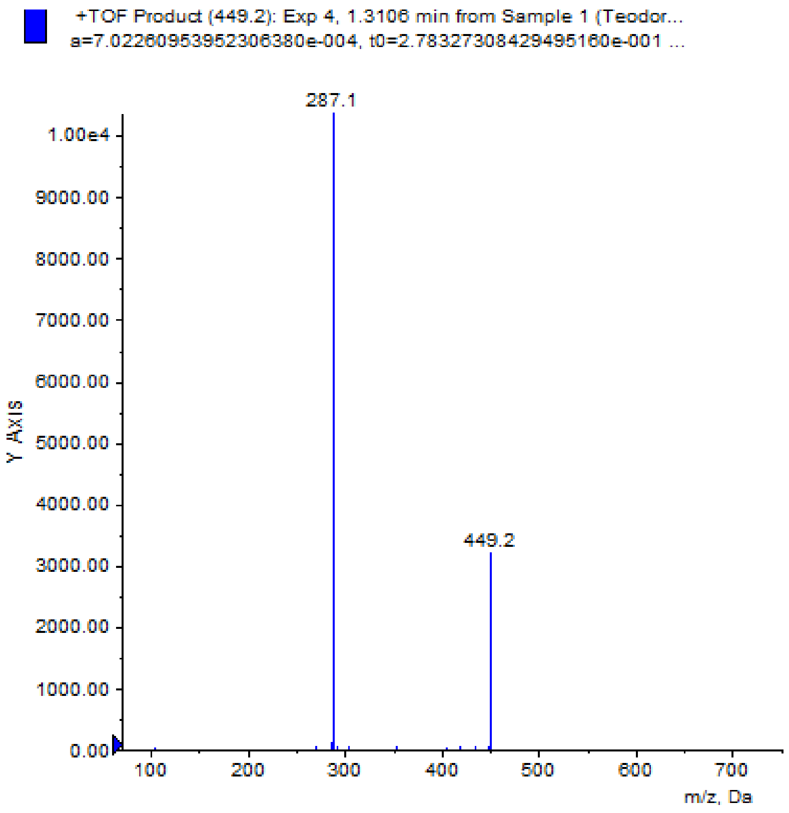

Delphinidin 3-glucoside in M 15

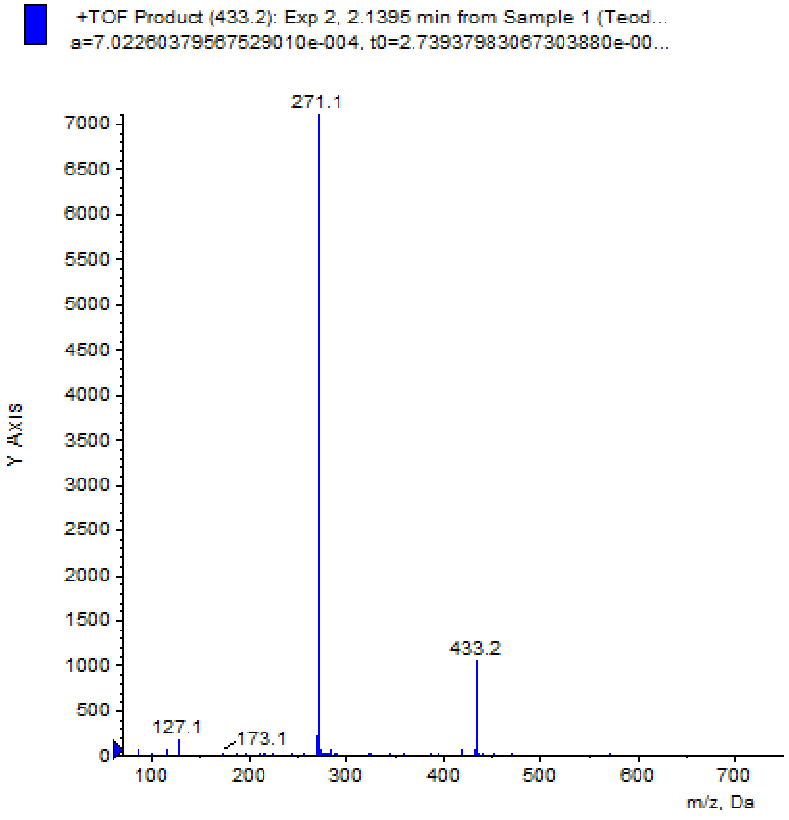

Pelargonidin 3-glucoside in M 15

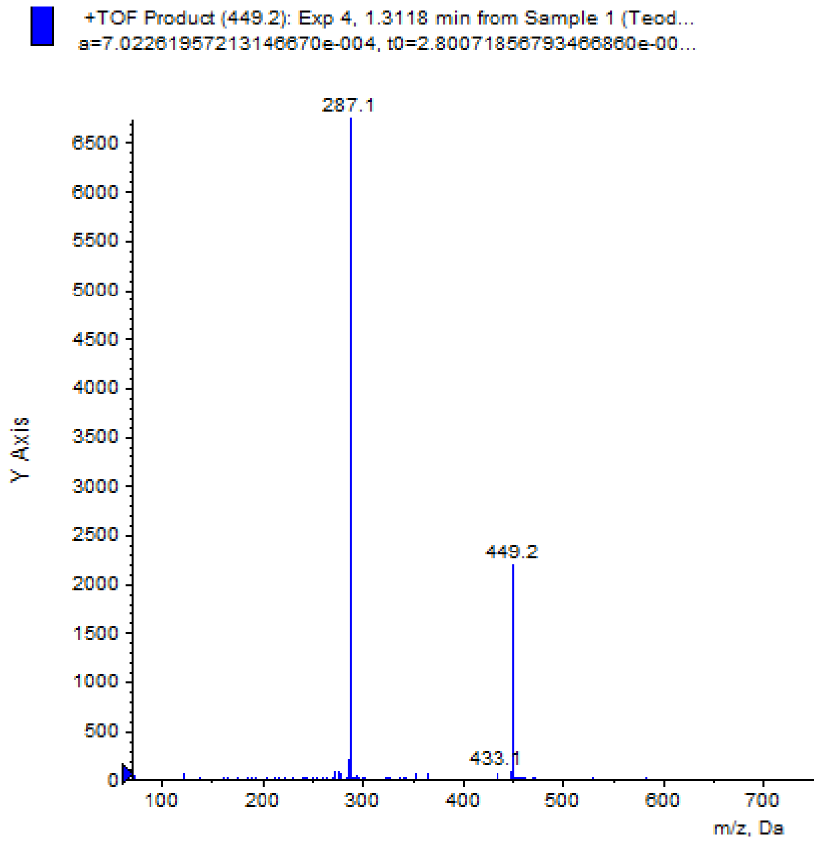

Cyanidin 3-glucoside in M 17

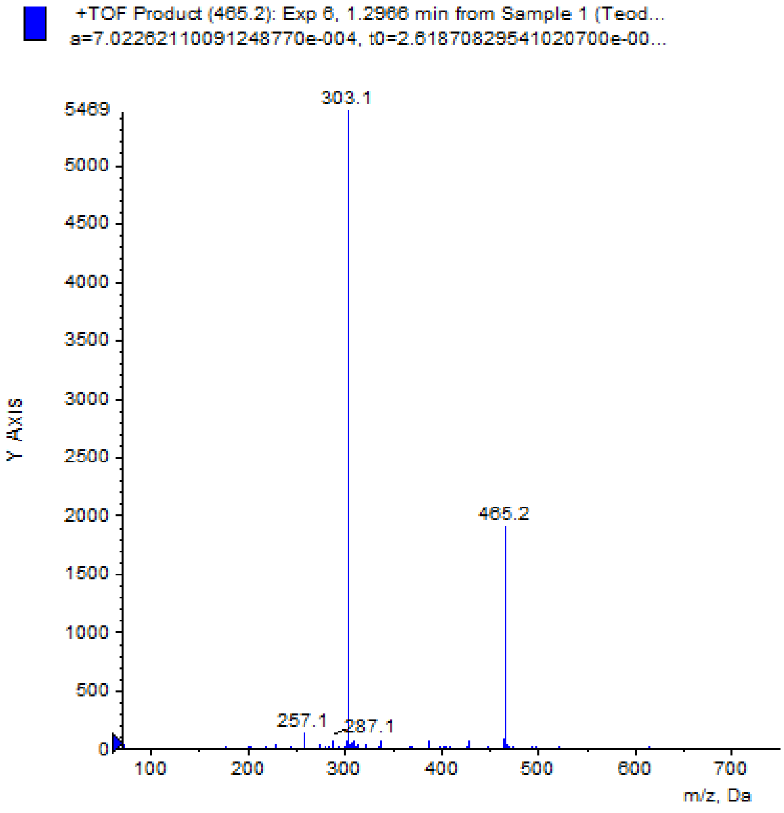

Delphinidin 3-glucoside in M 17

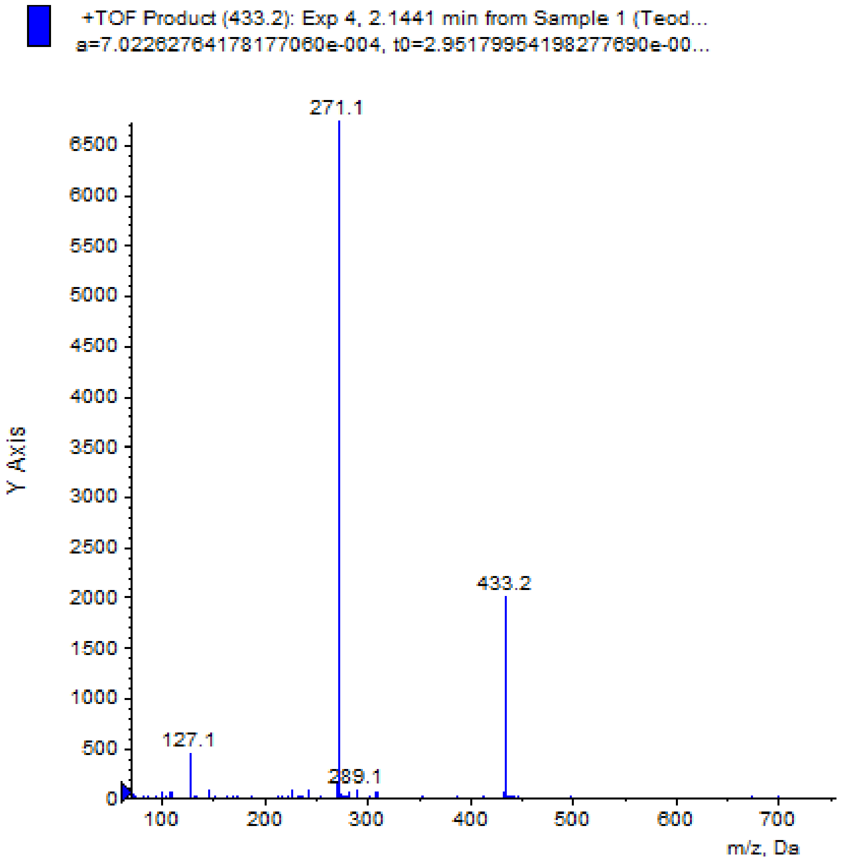

Pelargonidin 3-glucoside in M 17

Figure S4: MS/MS data concerning anthocyanin availability in M 2.3; M 15; M 17 mutant lines detected by QTRAP 5500 (AB SCIEX, USA).
